# Feasibility of Calculating and Maintaining Near-Infrared Spectroscopy-Guided Personalized Mean Arterial Pressure Targets in Adults With Critical Illness: A Pilot Clinical Study

**DOI:** 10.1097/CCE.0000000000001383

**Published:** 2026-03-03

**Authors:** Jiale Xie, Jasmine M. Khan, David M. Maslove, John Muscedere, Stephanie Sibley, J. Gordon Boyd

**Affiliations:** 1 Translational Medicine Institute, Queen’s University, Kingston, ON, Canada.; 2 Centre of Neuroscience, Queen’s University, Kingston, ON, Canada.; 3 Department of Critical Care Medicine, Queen’s University, Kingston, ON, Canada.

**Keywords:** arterial pressure, cerebrovascular circulation, critical illness, delirium, feasibility studies, near-infrared spectroscopy

## Abstract

**OBJECTIVES:**

Critically ill patients have a high risk for delirium, which may result from inadequate cerebral perfusion. One resuscitation goal for adult critically ill patients is maintaining mean arterial pressure (MAP) greater than 65 mm Hg, regardless of diagnosis or patient characteristics. Recent data suggest a high degree of individual variability in optimal MAP (MAPopt) due, in part, to whether autoregulation is intact or absent. The overall objective of this study was to evaluate the feasibility of maintaining critically ill patients within an individualized MAPopt range identified noninvasively with near-infrared spectroscopy, a technology that measures regional cerebral oxygen saturation (rSo_2_).

**DESIGN:**

Pilot interventional feasibility study.

**SETTING:**

Mixed ICU at a tertiary hospital.

**PATIENTS:**

Sixteen adult critically ill patients were enrolled within 24 hours of ICU admission. Exclusion criteria included expected survival less than 24 hours, neurologic or neurosurgical diagnoses, absence of an arterial catheter, or pregnancy.

**INTERVENTIONS:**

MAP and rSo_2_ data were recorded for 24 hours and processed through a custom algorithm. A running correlation coefficient between MAP and rSo_2_ was generated. Periods where there was zero or negative correlation between MAP and rSo_2_ reflected intact autoregulation. The MAP range where this correlation was near zero was determined to be the MAPopt. Vasoactive medications were used to maintain patients within that target range for the next 48 hours.

**MEASUREMENTS AND MAIN RESULTS:**

The enrollment rate was 0.5 patients/mo (goal 1/mo). MAPopt was successfully identified in 12 patients (75%) and maintained for 61% ± 17% of the follow-up period. The proportion of time spent within MAPopt strongly correlated with the width of the MAPopt range (*r* = 0.729; *p* = 0.017). There were no adverse events associated with the intervention.

**CONCLUSIONS:**

Although enrollment was lower than expected, MAPopt was calculated in the majority of patients. Maintaining patients within individualized MAPopt ranges was challenging, particularly when this range was narrow.

**KEY POINTS**

**Question**: Can personalized mean arterial pressure (MAP) targets based on cerebral autoregulation be identified and implemented in real-time in the ICU?

**Findings**: Optimal MAP was successfully calculated in 75% of patients and maintained for 61% ± 17% of the intervention period.

**Meaning**: While individualized MAP targets can be identified, it is challenging to maintain patients within those targets, particularly when the optimal range is narrow.

Every year, approximately 150,000 Canadians survive an admission to an ICU (1). An estimated 60,000 of these survivors experience cognitive impairment equivalent to moderate traumatic brain injury (2). The cognitive impairment experienced by ICU survivors is associated with diminished quality of life and reduced employment (3, 4). Delirium, characterized by disorganized thinking, inattention, fluctuating mental state, or consciousness (5), is a key and potentially modifiable risk factor for long-term cognitive dysfunction in ICU survivors (2, 5). Among ICU patients, those in shock, and/or require mechanical ventilation are at particularly high risk for developing delirium (6).

Poor cerebral perfusion has been suggested as a key determinant of delirium (7, 8). Correspondingly, there is growing evidence suggesting cerebral autoregulation, the physiologic mechanism that ensures stable blood flow to the brain across a range of systemic blood pressures (BPs), is impaired in critically ill patients with delirium (9, 10). When BP falls outside an individual’s autoregulatory range, cerebral blood flow becomes pressure-passive, increasing the risk of hypoperfusion or hyperemia. Cerebral autoregulation can be assessed noninvasively by correlating regional cerebral oxygen saturation (rSo_2_) with mean arterial pressure (MAP) over the span of min/s. The cerebral autoregulation index (COx) is the correlation coefficient between rSo_2_ and MAP (9, 10). As MAP fluctuates, a significantly positive COx reflects a passive relationship between MAP and rSo_2_, reflecting dysfunctional cerebral autoregulation. Conversely, a COx value near or at zero means that rSo_2_ remains relatively constant despite fluctuations in MAP, suggesting that cerebral autoregulation is intact. The MAP range where COx is at (or near) zero is termed the optimal MAP (MAPopt), which represents the individualized MAP target for that patient. It is hypothesized that deviation from MAPopt is associated with delirium. Several observational studies in patients undergoing cardiac surgery and in the ICU found that deviation from MAPopt, either intraoperatively or during the early ICU stay, measured as time or area outside MAPopt, was associated with the incidence and/or duration of delirium (9, 11–13). Furthermore, one randomized control trial in cardiac surgery found that keeping patients in their MAPopt intraoperatively significantly decreased the incidence of postoperative delirium (14).

rSo_2_ can be measured non-invasively via near-infrared spectroscopy (NIRS) and high-fidelity MAP data are routinely collected in ICU patients with an arterial catheter. Prior studies have demonstrated that individualized MAP targets can be retrospectively calculated, with substantial interindividual variability observed (15, 16). More recently, Peng et al (17) successfully identified and targeted MAPopt in patients with septic shock, although targeting was only maintained until shock stabilization. However, whether MAPopt can be feasibly calculated and sustained across a broader, mixed population of critically ill ICU patients for longer durations remains unknown.

This pilot study aimed to assess the feasibility of calculating MAPopt and maintaining MAP within that range in real-time for critically ill patients. Secondary objectives included exploring the incidence and duration of delirium, area and time spent with autoregulatory dysfunction, and the associations between autoregulatory dysfunction and delirium in this cohort.

## MATERIAL AND METHODS

### Patient Enrollment

Enrollment occurred from July 2022 to February 2025 in a level-3 mixed medical-surgical-trauma-neurosciences ICU in Kingston, Canada. All study procedures were reviewed by Queen’s Health Sciences and Affiliated Teaching Hospitals Research Ethics Board (DMED-2489-21). A 24 hours deferred consent model was used. The trial was registered on clinicaltrials.gov (NCT07296029). All procedures were followed in accordance the Helsinki Declaration of 1975. We enrolled patients (≥ 18 yr) who had respiratory failure, defined as requiring invasive mechanical ventilation for an expected duration of more than 24 hours, and/or experienced shock of any etiology. This inclusion strategy follows prior studies focused on respiratory failure and shock and captures a population at especially high risk for delirium. Exclusion criteria included: 1) greater than 24 hours since ICU admission, 2) life expectancy less than 24 hours, 3) a primary CNS admitting diagnosis (e.g., traumatic brain injury, stroke), and 4) pregnancy. All patients admitted to the ICU were screened daily, Monday to Friday, 9 am to 4 pm, for eligibility by a research coordinator and/or the principal investigator.

### MAPopt Data Capture and Calculation

**Figure 1.**
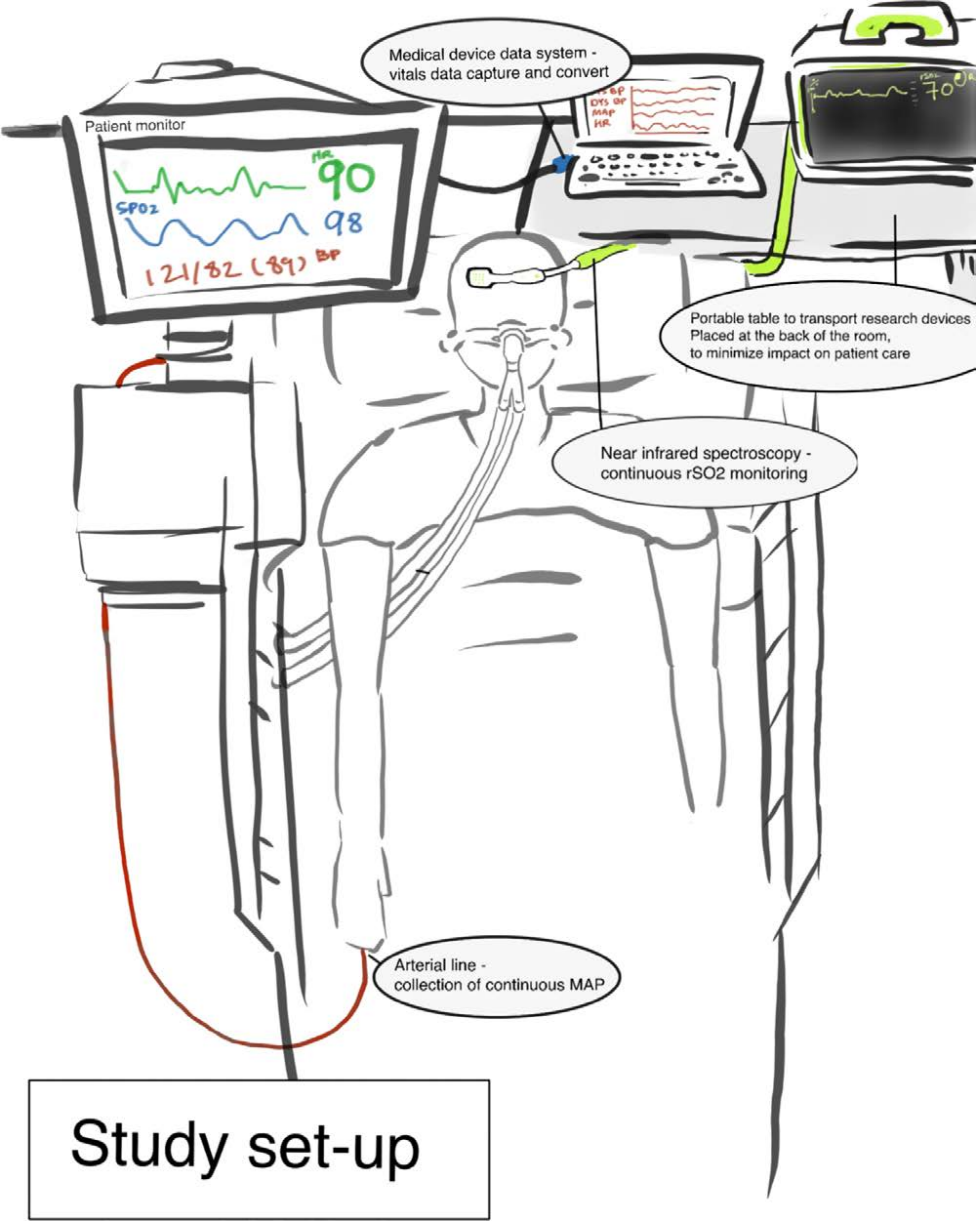
An illustration of a typical study setup. BP = blood pressure, MAP = mean arterial pressure, rSo_2_ = regional cerebral oxygen saturation, Spo_2_ = peripheral oxygen saturation.

To obtain rSo_2_ and MAP data necessary for MAPopt calculation, a FORESIGHT Elite cerebral oximeter (Edwards Lifesciences, Irvine, CA) was setup at the patient’s bedside to capture rSo_2_. MAP data was extracted from the bedside monitors using commercially available software and hardware (Medicollector, Boston, MA; **Fig. 1**). After 24 hours, a study team member entered the rSo_2_ and MAP data through a custom-written algorithm that cleans extracted MAP data and allowed for real-time interpretation of rSo_2_-MAP relationships to compute MAPopt (15, 16, 18). This is the same algorithm used previously to establish an association between dysfunctional autoregulation and delirium in a similar patient population (9). Autoregulation was assessed by calculating COx, the moving correlation between rSo_2_ and MAP within 30-minute windows advanced every minute. COx values were then correlated with MAP and MAP values were binned according to their corresponding COx index. MAPopt was defined as the average MAP within the COx bin centered at 0 (0 ± 1 sd). Autoregulation limits were defined as the MAP values corresponding to COx values 1 sd above and below zero. For the first 24 hours, data were collected, and at the end of this period, cerebral autoregulation status and the MAPopt range was determined. For the next 48 hours, each patient’s MAPopt was communicated to the bedside care team, and MAPopt was targeted using norepinephrine (dose range 1–10 µg/min) and/or labetalol infusions (dose range 0–3 mg/min). As these treatments are common in a critical care setting, no serious adverse effects were anticipated beyond the known side effects of these interventions. Patients were closely monitored in the ICU, and any adverse events were immediately reported to both the study and clinical teams, prompting a protocolized de-escalation of treatment if necessary.

### Outcome Data Collection

Primary feasibility outcomes included: enrollment rate (patients/mo), proportion of patients for which MAPopt could be calculated (%), proportion of time outside of MAPopt for the 48-hour intervention period (time outside MAPopt/[time within MAPopt + time outside MAPopt] × 100%), and adverse event rate (as defined in Good Clinical Practice guidelines).

Secondary outcomes included the percent time with impaired autoregulation, area outside of MAPopt (defined as the product of deviation magnitude and duration), and the incidence and duration of delirium. Duration of dysfunctional autoregulation was captured through NIRS and arterial catheter data. Area outside of MAPopt was monitored using the arterial catheter data throughout the 48 hours of intervention. Delirium was captured through daily screening using the Confusion Assessment Method for the ICU (CAM-ICU) and brief CAM (once on the hospital ward) by a trained study team member for up to 30 days of participants’ hospital stay. Although sedative medications were not specifically held for the CAM-ICU assessment, as per our institutional policy, patients receive an analgesia-first strategy, with a target sedation level of a Richmond Agitation-Sedation Scale (RASS) of 0 to –1. Coma was defined as a RASS of –4/–5. Clinical and demographic data were collected from the electronic medical record (EMR). As these patients were part of a broad program of research (Cerebral Oxygenation and Neurological Outcomes Following Critical Illness; CONFOCAL-2 [NCT03141619 ClinicalTrials.gov]), variables associated with delirium were also collected. These included demographic risk factors for delirium, such as history of hypertension and alcohol use disorder, severity of illness (Acute Physiology and Chronic Health Evaluation II), and benzodiazepine and opioid use.

### Statistical Analysis

Feasibility was defined a priori as the ability to: 1) enroll greater than or equal to 1 patient per month; 2) obtain informed consent in greater than 80% of eligible patients; 3) determine MAPopt targets in greater than 75% of patients; 4) maintain target MAP within the target range for greater than 90% of the time; and 5) assess the safety of using this alternative MAP resuscitation target. Custom R code was written to determine whether MAP was within the target range (code available on https://github.com/jiale-xie/MAPopt/blob/master/mapopt_time&auc_analysis.Rmd). Values generated were charted and reported as descriptive statistics.

Directionality and statistical significance of COx values were used to evaluate disturbed autoregulation as described previously (9). Briefly, time-rolling Spearman rho correlation coefficients were calculated between rSo_2_ and MAP over the monitoring duration. Positive COx values that were statistically significant (*p* < 0.0001) were indicators of disturbed autoregulation. Negative or zero values were indicators of intact cerebral autoregulation. Exploratory analyses were conducted using Spearman correlation to examine the associations between the percentage of time spent with dysfunctional autoregulation and delirium, as well as between area outside of MAPopt and delirium. Analysis was performed via Excel (Microscoft, Redmond, WA), R (R Studio, Boston, MA), Python (Wilmington, DE), and SPSS (IBM Corp, Armonk, NY).

## RESULTS

### Population Characteristics

Sixteen participants were enrolled over 32 months (from July 2022 to February 2025). Their demographic information is presented in **Table 1**. The median age of the cohort was 58 and the sex distribution was balanced. Most participants were admitted primary due to a respiratory diagnosis or sepsis. In their first 24 hours of ICU admissions, all participants required vasopressor support and 15 of 16 (94%) were mechanically ventilated.

**TABLE 1.** Baseline Characteristics and Clinical Data for Enrolled Patients

Clinical/Demographic Feature	Enrolled Patients (*n* = 16)
Age, yr, median (IQR)	68 (58–75)
Male, *n* (%)	9 (56)
Weight (kg), median (IQR)	81 (68–93)
Height (cm), median (IQR)	170 (160–175)
Comorbidities, *n* (%)	
Alcohol use disorder	2 (12)
Hypertension	11 (69)
Admitting diagnosis, *n* (%)	
Respiratory	9 (56)
Sepsis	4 (25)
Gastrointestinal	1 (6)
Trauma	1 (6)
Other	1 (6)
Clinical Frailty Scale^a^, median (IQR)	4 (3–5)
Acute Physiology and Chronic Health Evaluation II^b^, median (IQR)	19 (17–22)
Vasopressor support first 24 hr of ICU admission (%)	16 (100)
Mechanically ventilated first 24 hr of ICU admission (%)	15 (94)
Pao_2_/Fio_2_ ratio, median (IQR)	213 (173–264)
Total norepinephrine dose^c^, µg, median (IQR)	14,940 (5,661–18,870)
Total vasopressin dose^c^, U, median (IQR)	0 (0–43.7)
Total labetalol dose^c^, mg, median (range)	0 (0–1,260)
Surgery in first 24 hr, *n* (%)	7 (44)
Delirium^d^, *n* (%)	11 (69)
ICU length of stay, d, median (IQR)	11 (4–29)
ICU mortality, *n* (%)	6 (38)

IQR = interquartile range.

a The Clinical Frailty Scale is used as a measure of preexisting frailty.

b The Acute Physiology and Chronic Health Evaluation II score is used as a measure of illness severity in the ICU.

c Total dose of each agent received by each participant during the 72-hr monitoring period.

d Confusion Assessment Method positive at least once during the hospital stay.

### Primary Outcomes: Feasibility

#### Enrollment

**Figure 2.**
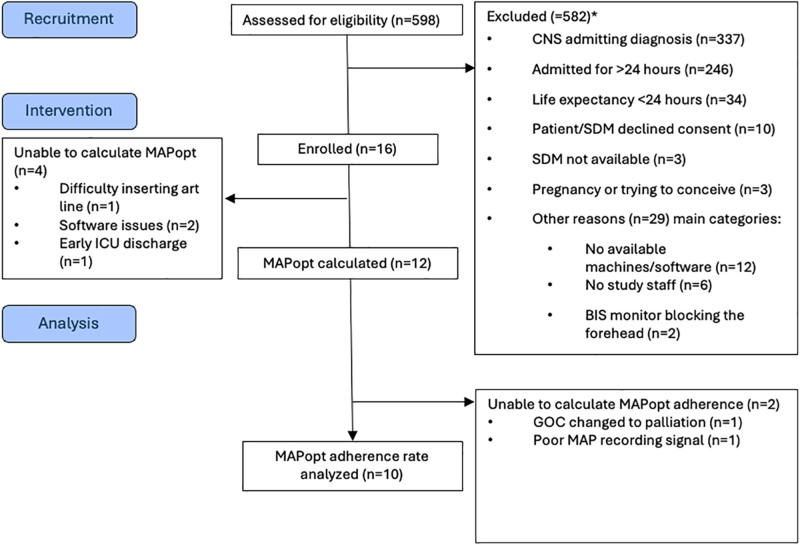
Modified Consolodated Standard of Reporting Trials diagram. *These exclusion criteria are not mutually exclusive. Some patients approached had met several exclusion criteria. Software issues concern the mean arterial pressure (MAP) recordings. These include software shutdown while recording and interested values not selected when setting up the recording. BIS = bispectral index, GOC = goals of care, MAPopt = optimal mean arterial pressure, SDM = substitute decision-maker.

The average enrollment rate was 0.5 patients per month. Among all participants that were screened, ten declined to participate (2%; **Fig. 2**). Among all participants approached ten of 26 (38%) declined consent.

#### MAPopt Identification

MAPopt could be identified in 12 of 16 patients (75%) within 48 hours of ICU admission (**Table 2** includes each participant’s MAPopt values). Barriers included: difficulty with arterial catheter insertion (*n* = 1), missing MAP data due to software issues (*n* = 2), and early ICU discharge before the intervention period (*n* = 1).

**TABLE 2.** Participant’s Optimal Mean Arterial Pressure Range and the Percentage of Time They Spent Within Their Optimal Mean Arterial Pressure Range During the 48 Hours Intervention Period

Participant ID	Lower Limit of MAPopt (mm Hg)	Upper Limit of MAPopt (mm Hg)	MAPopt Range Width (mm Hg)	MAP Monitoring Duration (min) for Follow-Up	% Time Inside MAPopt (48-hr Intervention)
PI-001	68	77	9	1875	50%
PI-002	70	76	6	2883	28%
PI-005	65	77	12	Not recorded	Not recorded
PI-006	72	84	12	2966	46%
PI-007	72	85	14	1743	61%
PI-008	70	86	16	2883	73%
PI-010	67	84	17	2879	89%
PI-011	66	78	12	2888	70%
PI-012	57	64	7	1252	58%
PI-013	74	83	9	2466	64%
PI-014	69	79	10	1719	67%
PI-016	63	80	17	Not recorded	Not recorded
Mean (sd)	68 (5)	79 (6)	12 (4)	2355 (644)	61 (17)

ID = identification, MAP = mean arterial pressure, MAPopt = optimal MAP.

MAPopt could not be calculated for PI-003, PI-004, PI-009, and PI-015.

Forty-eight hr MAPopt monitoring not possible for PI-05 or PI-16 because of poor signal recording quality and a change in patient goals of care to palliation, respectively.

#### MAPopt Implementation

**Figure 3.**
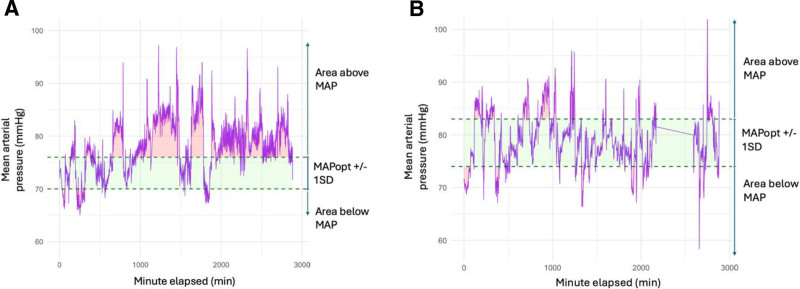
Examples of arterial line recordings from two illustrative patients during the 48-hr follow-up period. The mean arterial pressure (MAP) tracing is shown in *purple*. The optimal MAP (MAPopt) range is *shaded* in *green*, and area outside of this range are *shaded* in *red*. **A**, Illustrative participant with more area above than below MAPopt. **B**, Illustrative participant with similar area below and above MAPopt.

**Figure 4.**
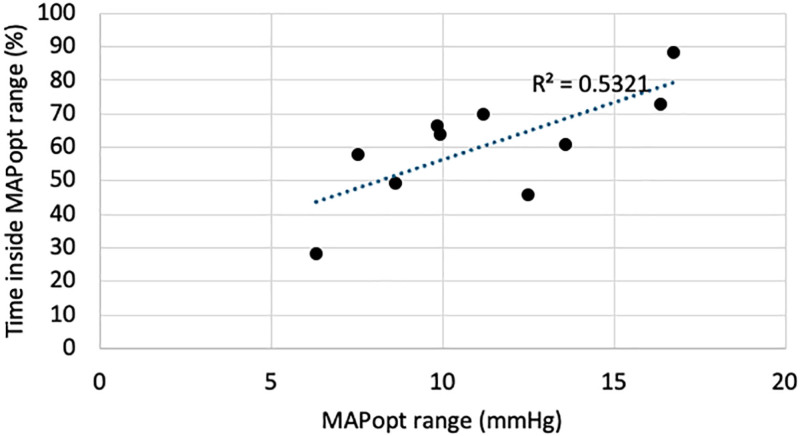
*Scatter plot* demonstrating the association between participants’ optimal mean arterial pressure (MAPopt) range width and the percentage of time they spent within their MAPopt range during the 48 hr follow-up period. The *solid line* represents a linear trendline (line of best fit), calculated using the least-squares method in Microsoft Excel. The *R*^ 2^ value is displayed to indicate goodness of fit. Pearson correlation coefficient: 0.729; *p* = 0.017; *n* = 10.

Among patients for whom MAPopt could be determined, time spent within MAPopt during the follow-up period ranged from 28% to 89% (mean = 61%, sd = 17%; Table 2 includes the percentage of time each participant spent in their MAPopt range). Example physiologic recordings of two illustrative patients are shown in **Figure 3**. The ability to maintain patients in their MAPopt range was highly correlated with the width of their range (**Fig. 4**; Pearson *r* [8] = 0.729; *p* = 0.017). No adverse events were reported; thus, no protocolized de-escalation of any intervention was required.

### Adverse Events

No patient safety incidents were associated with the intervention. Seven patients died from causes related to their admission diagnoses, and none were attributed to the intervention.

### Secondary Outcomes

#### Delirium

Daily delirium screening was completed by the research team on 95% of eligible days. The distribution of patients’ delirium status on each day following initial study enrollment is shown in **Supplemental Figure 1** (https://links.lww.com/CCX/B605).

#### Delirium and Autoregulation Dysfunction

**Figure 5.**
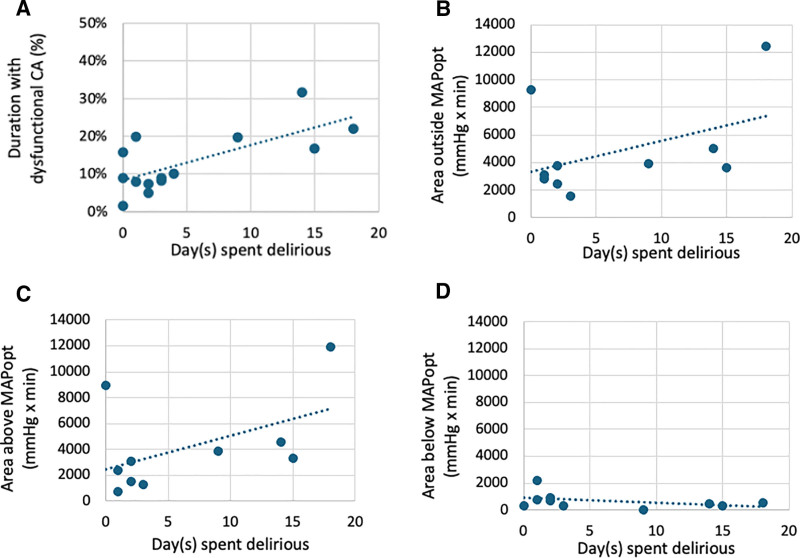
*Scatter plots* showing the association between cerebral autoregulation (CA) metrics and days spent delirious. *Each plot* displays individual data points with a fitted linear regression line. The *x*-axis represents the number of days spent delirious, and the *y*-axis represents a given CA metric: **A**, duration of disturbed autoregulation; **B**, area outside optimal mean arterial pressure (MAPopt); **C**, area above MAPopt; and **D**, area below MAPopt. A significant positive association was observed in **A** (ρ = 0.571, *p* = 0.033), while no significant associations were observed in **B–D** (*p* > 0.1 for all).

As an exploratory analysis, we examined whether there was an association between the duration of disturbed cerebral autoregulation and delirium. As shown in **Figure 5*A***, there was a significant correlation between the duration on disturbed autoregulation and the number of days a patient experienced delirium (Spearman ρ = 0.571; *p* = 0.033; *n* = 14) (Include partial recordings [PI-05, PI-09, PI-15, and PI-16] for whom the cerebral autoregulation index recording may be missed for the first 24 hr or during the follow-up periods. Inaccurate recording portions were removed and only accurate recording portions from the 72 hr study duration were included in the analysis.) However, as shown in **Figure 5*B***, there was no significant correlation between area outside MAPopt and delirium days (ρ = 0.293; *p* = 0.412; *n* = 10). Likewise, there was no significant correlation with the area above (**Fig. 5*C***; ρ = 0.416; *p* = 0.233; *n* = 10) nor the area below (**Fig. 5*D***; ρ = –0.433; *p* = 0.211; *n* = 10) and the number of delirium days.

## DISCUSSION

Common resuscitation targets for critically ill patients include a MAP goal of greater than 65 mm Hg. However, emerging evidence indicates that cerebral autoregulation capacity varies among patients, suggesting that personalized MAP targets could be identified and targeted, potentially reducing complications such as delirium and cognitive dysfunction. This pilot study explored the feasibility of implementing individualized MAP targets based on noninvasive real-time cerebral autoregulation monitoring. Our results demonstrate that real-time MAPopt calculation is feasible in most patients. However, maintaining BP within personalized ranges was challenging, particularly when those ranges were narrow.

This pilot highlighted several practical insights that may be helpful for future investigations. Importantly, enrollment rates were lower than anticipated. This was multifactorial in nature. First, this was due to the exclusion of patients admitted on weekends and limited staff availability. Approximately 40% of patients screened were excluded because they had been admitted for more than 24 hours, with weekend admissions being a primary reason. Expanding recruitment to weekends and training additional personnel to perform MAPopt calculations may improve accrual. Remote access to NIRS and MAP data could support the calculation of MAPopt during off-hours or while staff members are unable to attend in person. Second, arterial line availability and data quality also posed challenges; some eligible participants were excluded due to the lack of invasive monitoring or recording errors. Given this constraint, future studies could explore noninvasive continuous BP monitoring technologies (e.g., Finapres, The Netherlands). Prior studies have demonstrated the feasibility of calculating MAPopt from noninvasive signals (19, 20), and one found an association between autoregulation dysfunction and 3-month mortality using Finometer-derived data (19). However, further work is needed to improve the quality of noninvasive BP recordings and study their association with outcomes, as available studies show that correlation indices and MAPopt calculated using these two methods do not always agree (20). Finally, nearly 60% of patients screened were excluded from our study because they had a primary CNS admitting diagnosis. While this decreased our overall enrollment rate, these patients were excluded because delirium may be more challenging to assess (21), and MAPopt ranges may be higher (22).

Data loss due to software errors, disconnection from bedside monitors, or interruptions from clinical care (e.g., room transfers, procedures) represented another key barrier. In our study, devices were checked 1–2 times per day; increasing monitoring frequency and incorporating simple visual indicators (e.g., bedside infographics identifying working vs. not working display) may improve signal continuity. Enhancing trial awareness and engagement among bedside teams, through active strategies such as personal discussions and EMR flags/alerts, and passive approaches like whiteboard reminders, departmental seminars, and newsletters may further promote protocol adherence (23–25).

The observed variability in the maintaining MAP targets highlights the complexity of BP management in the ICU. Unlike in the operating room, where strict intraoperative BP control may be more feasible (26), ICU clinicians face competing priorities and fluctuating physiologic demands. Five patients in our cohort had autoregulatory ranges less than or equal to 10 mm Hg, rendering tight adherence particularly difficult. Individual vascular reactivity, sedation depth, multiple drug infusions, dynamic changes in Co_2_ tension, and patient-specific pharmacologic responsiveness all contribute to this variability (27). Future protocols might explore broader autoregulatory ranges (e.g., ± 1.5 or 2 sd from MAPopt), targets focused on lower limits alone (28), or a more focused intervention duration. A recent study (17) has applied individualized MAP targets until shock resolution criteria were met, which typically ranges from a few hours to a day. Another potential avenue is to develop MAPopt targets that account for other physiologic variables impacting perfusion. One study that used a correlation threshold to determine MAPopt adjusted their threshold based on participant end-tidal co_2_ level (29). These modifications may enhance the feasibility of MAPopt protocol in the ICU.

We observed a preliminary association between dysfunctional autoregulation and ICU delirium, consistent with prior studies suggesting that impaired autoregulation may compromise cerebral perfusion and contribute to delirium. Both observational and randomized controlled studies have reported links between impaired autoregulation and delirium in critically ill populations, including patients undergoing cardiac surgery (14, 30) and sepsis (9, 17, 31–33). This association has also been highlighted in two systematic reviews, although the strength of conclusions was limited by methodological heterogeneity in study designs and autoregulation assessment methods (34, 35). While our study was not powered to confirm this relationship definitively, our findings reinforce the notion that dysfunctional cerebral autoregulation may be an important contributor to, or mediator in, the pathophysiology of delirium in critically ill adults.

This pilot is one of the first to evaluate the feasibility of individualized MAP titration guided by continuous cerebral autoregulation monitoring in critically ill adults without primary brain injury in the ICU setting (17). While limited by small sample size, incomplete data capture, and the exclusion of patients with primary brain injury (who may have distinct autoregulatory profiles and different MAPopt thresholds), this study offers valuable proof-of-concept data. Follow-up recording was not possible for two participants and was short in five others due to procedural interruptions or care transitions. Signal quality was further affected by limitations inherent to NIRS-based technology, including assumptions about tissue oxygen diffusion and susceptibility to motion artifacts or extracerebral contamination (18, 36). These constraints may affect both internal validity and generalizability. Nonetheless, our findings provide a foundational framework for larger, more definitive trials examining whether individualized hemodynamic management based on cerebral autoregulation can meaningfully improve neurologic outcomes in critically ill populations.

## CONCLUSIONS

This pilot study has identified challenges and opportunities for identifying personalized MAP targets using NIRS-based cerebral autoregulation monitoring in critically ill patients without acute brain injury. MAPopt could be determined in most patients, although challenges with signal quality and data completeness were noted. Maintaining MAP within the individualized target range was especially difficult in patients with a narrow MAPopt range. These findings highlight the need to enhance data capture potentially through exploring alternatives to arterial line monitoring, improve trial communication and adherence strategies, and refine target calculation methods. Insights from this study will inform future trials evaluating the clinical utility of autoregulation-guided BP management in reducing delirium and other neurologic complications in the ICU.

## ACKNOWLEDGMENTS

We thank our research coordinators, Ms. Michaela Caldwell, Ms. Tracy Boyd, and Ms. Miranda Hunt. Also, we thank Drs. Robert Fowler and Bruno Ferreryo from the Canadian Critical Care Trials Group for their valuable feedback as part of the group’s internal peer review process.

The Canadian Critical Care Trials Group and the Cerebral Oxygenation and Neurological Outcomes Following Critical Illness (CONFOCAL) Research Program members are Michael Wood, Kevin Lee, Miranda Hunt, Michaela Hanley, and Tracy Boyd.

## Supplementary Material



## References

[1] Canadian Institute for Health Information: Care in Canadian ICUs. 2016. Available at: https://secure.cihi.ca/free_products/ICU_Report_EN.pdf. September 15, 2025

[2] Pandharipande PP Girard TD Jackson JC; BRAIN-ICU Study Investigators: Long-term cognitive impairment after critical illness. N Engl J Med 2013; 369:1306–1316 24088092 10.1056/NEJMoa1301372 PMC3922401

[3] Norman BC Jackson JC Graves JA: Employment outcomes after critical illness: An analysis of the bringing to light the risk factors and incidence of neuropsychological dysfunction in ICU survivors cohort. Crit Care Med 2016; 44:2003–2009 27171492 10.1097/CCM.0000000000001849 PMC5069078

[4] Iwashyna TJ Ely EW Smith DM: Long-term cognitive impairment and functional disability among survivors of severe sepsis. JAMA 2010; 304:1787–1794 20978258 10.1001/jama.2010.1553 PMC3345288

[5] Barr J Fraser GL Puntillo K; American College of Critical Care Medicine: Clinical practice guidelines for the management of pain, agitation, and delirium in adult patients in the intensive care unit. Crit Care Med 2013; 41:263–306 23269131 10.1097/CCM.0b013e3182783b72

[6] Honarmand K Lalli RS Priestap F: Natural history of cognitive impairment in critical illness survivors. A systematic review. Am J Respir Crit Care Med 2020; 202:193–201 32078780 10.1164/rccm.201904-0816CI PMC7365360

[7] Maldonado JR: Acute brain failure: Pathophysiology, diagnosis, management, and sequelae of delirium. Crit Care Clin 2017; 33:461–519 28601132 10.1016/j.ccc.2017.03.013

[8] Pfister D Siegemund M Dell-Kuster S: Cerebral perfusion in sepsis-associated delirium. Crit Care 2008; 12:R63 18457586 10.1186/cc6891 PMC2481444

[9] Lee KF Wood MD Maslove DM: Dysfunctional cerebral autoregulation is associated with delirium in critically ill adults. J Cereb Blood Flow Metab 2019; 39:2512–2520 30295556 10.1177/0271678X18803081 PMC6893984

[10] Rivera-Lara L Zorrilla-Vaca A Geocadin RG: Cerebral autoregulation-oriented therapy at the bedside: A comprehensive review. Anesthesiology 2017; 126:1187–1199 28383324 10.1097/ALN.0000000000001625

[11] Hori D Max L Laflam A: Blood pressure deviations from optimal mean arterial pressure during cardiac surgery measured with a novel monitor of cerebral blood flow and risk for perioperative delirium: A pilot study. J Cardiothorac Vasc Anesth 2016; 30:606–612 27321787 10.1053/j.jvca.2016.01.012 PMC5508106

[12] Hori D Brown C Ono M: Arterial pressure above the upper cerebral autoregulation limit during cardiopulmonary bypass is associated with postoperative delirium. Br J Anaesth 2014; 113:1009–1017 25256545 10.1093/bja/aeu319 PMC4235573

[13] Nakano M Nomura Y Whitman G: Cerebral autoregulation in the operating room and intensive care unit after cardiac surgery. Br J Anaesth 2021; 126:967–974 33741137 10.1016/j.bja.2020.12.043 PMC8132879

[14] Brown CH IV Neufeld KJ Tian J: Effect of targeting mean arterial pressure during cardiopulmonary bypass by monitoring cerebral autoregulation on postsurgical delirium among older patients: A nested randomized clinical trial. JAMA Surgery 2019; 154:819–826 31116358 10.1001/jamasurg.2019.1163 PMC6537779

[15] Khan JM Wood MD Lee KFH: Delirium, cerebral perfusion, and high-frequency vital-sign monitoring in the critically ill. The CONFOCAL-2 feasibility study. Ann Am Thorac Soc 2021; 18:112–121 32780600 10.1513/AnnalsATS.202002-093OC

[16] Khan JM Shore A Lee KFH: Cerebral autoregulation-based mean arterial pressure targets and delirium in critically ill adults without brain injury: A retrospective cohort study. Can J Anaesth 2023; 71:107–117 37932650 10.1007/s12630-023-02609-w

[17] Peng Q Liu X Ai M: Cerebral autoregulation-directed optimal blood pressure management reduced the risk of delirium in patients with septic shock. J Intensive Med 2024; 4:376–383 39035614 10.1016/j.jointm.2023.12.003 PMC11258506

[18] Khan JM Maslove DM Boyd JG: Optimized arterial line artifact identification algorithm cleans high-frequency arterial line data with high accuracy in critically ill patients. Crit Care Explor 2022; 4:e0814 36567784 10.1097/CCE.0000000000000814 PMC9762921

[19] Pham P Bindra J Chuan A: Are changes in cerebrovascular autoregulation following cardiac arrest associated with neurological outcome? Results of a pilot study. Resuscitation 2015; 96:192–198 26316278 10.1016/j.resuscitation.2015.08.007

[20] Bindra J Pham P Aneman A: Non-invasive monitoring of dynamic cerebrovascular autoregulation using near infrared spectroscopy and the finometer photoplethysmograph. Neurocrit Care 2016; 24:442–447 26490778 10.1007/s12028-015-0200-3

[21] von Hofen-Hohloch J Awissus C Fischer MM: Delirium screening in neurocritical care and stroke unit patients: A pilot study on the influence of neurological deficits on CAM-ICU and ICDSC outcome. Neurocrit Care 2020; 33:708–717 32198728 10.1007/s12028-020-00938-y PMC7736013

[22] Xie J Carbonara AR Al-Battashi A-W: Individualized mean arterial pressure targets in critically ill patients guided by non-invasive cerebral-autoregulation: A scoping review. Crit Care 2025; 29:196 40380314 10.1186/s13054-025-05432-5 PMC12084981

[23] Dale C Fowler RA Adhikari NKJ: Implementation of a research awareness program in the critical care unit: Effects on families and clinicians. Intensive Crit Care Nurs 2010; 26:69–74 19864137 10.1016/j.iccn.2009.09.003

[24] Cook D Taneja S Krewulak K; Canadian Critical Care Trials Group and Canadian Clinical Research Network: Barriers, solutions, and opportunities for adapting critical care clinical trials in the COVID-19 pandemic. JAMA Netw Open 2024; 7:e2420458 38995645 10.1001/jamanetworkopen.2024.20458 PMC11245722

[25] Fogel DB: Factors associated with clinical trials that fail and opportunities for improving the likelihood of success: A review. Contemp Clin Trials Commun 2018; 11:156–164 30112460 10.1016/j.conctc.2018.08.001 PMC6092479

[26] Futier E Lefrant J-Y Guinot P-G; INPRESS Study Group: Effect of individualized vs standard blood pressure management strategies on postoperative organ dysfunction among high-risk patients undergoing major surgery: A randomized clinical trial. JAMA 2017; 318:1346–1357 28973220 10.1001/jama.2017.14172 PMC5710560

[27] Claassen JAHR Thijssen DHJ Panerai RB: Regulation of cerebral blood flow in humans: Physiology and clinical implications of autoregulation. Physiol Rev 2021; 101:1487–1559 33769101 10.1152/physrev.00022.2020 PMC8576366

[28] Liu X Akiyoshi K Nakano M: Determining thresholds for three indices of autoregulation to identify the lower limit of autoregulation during cardiac surgery. Crit Care Med 2021; 49:650–660 33278074 10.1097/CCM.0000000000004737 PMC7979429

[29] Goettel N Patet C Rossi A: Monitoring of cerebral blood flow autoregulation in adults undergoing sevoflurane anesthesia: A prospective cohort study of two age groups. J Clin Monit Comput 2016; 30:255–264 26285741 10.1007/s10877-015-9754-z

[30] Caldas JR Panerai RB Bor-Seng-Shu E: Dynamic cerebral autoregulation: A marker of post-operative delirium? Clin Neurophysiol 2019; 130:101–108 30503909 10.1016/j.clinph.2018.11.008 PMC7106549

[31] Schramm P Klein KU Falkenberg L: Impaired cerebrovascular autoregulation in patients with severe sepsis and sepsis-associated delirium. Crit Care 2012; 16:R181 23036135 10.1186/cc11665 PMC3682283

[32] Crippa IA Subirà C Vincent J-L: Impaired cerebral autoregulation is associated with brain dysfunction in patients with sepsis. Crit Care 2018; 22:327 30514349 10.1186/s13054-018-2258-8 PMC6280405

[33] Feng Q Ai M Huang L: Relationship between cerebral hemodynamics, tissue oxygen saturation, and delirium in patients with septic shock: A pilot observational cohort study. Front Med 2021; 8:641104 10.3389/fmed.2021.641104 PMC8660998 34901041

[34] Caldas JR Haunton VJ Panerai RB: Cerebral autoregulation in cardiopulmonary bypass surgery: A systematic review. Interact Cardiovasc Thorac Surg 2018; 26:494–503 29155938 10.1093/icvts/ivx357

[35] Longhitano Y Iannuzzi F Bonatti G: Cerebral autoregulation in non-brain injured patients: A systematic review. Front Neurol 2021; 12:732176 34899560 10.3389/fneur.2021.732176 PMC8660115

[36] Bird JD MacLeod DB Griesdale DE: Shining a light on cerebral autoregulation: Are we anywhere near the truth?. J Cereb Blood Flow Metab 2024; 44:1057–1060 38603610 10.1177/0271678X241245488 PMC11318395

