# Supplementary material for: Feasibility of Calculating and Maintaining Near-Infrared Spectroscopy-Guided Personalized Mean Arterial Pressure Targets in Adults With Critical Illness: A Pilot Clinical Study
Source: Crit Care Explor. 2026 Mar 3;8(3):e1383. doi: 10.1097/CCE.0000000000001383 (PMC12959832; doi:10.1097/CCE.0000000000001383)
Supplement: Supplementary file 1 [file cc9-8-e1383-s001.pdf]

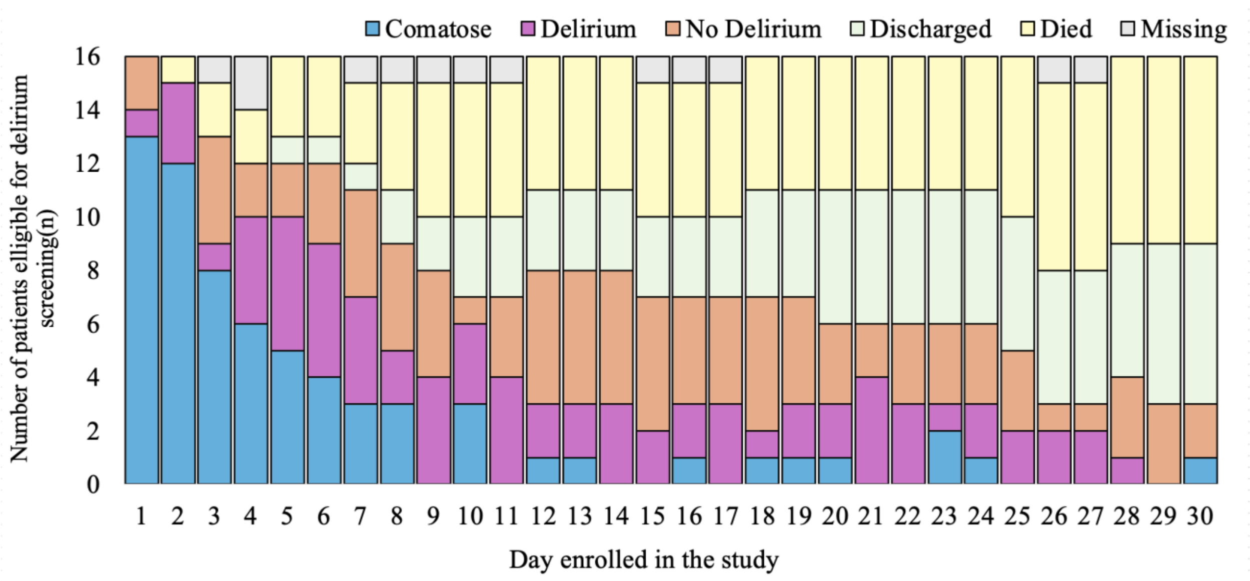

Supplemental Figure 1. Delirium status of all participants, categorized as comatose (defined as CAM-ICU "Unable to Assess,"), delirium, no delirium, died or discharged from the hospital, or assessment missed. The height of each bar represents the total number of patients assessed each day, and the segments within each bar indicate the proportion of patients with each corresponding delirium status.
